# CXCR5 induces perineural invasion of salivary adenoid cystic carcinoma by inhibiting microRNA-187

**DOI:** 10.18632/aging.203097

**Published:** 2021-06-10

**Authors:** Mei Zhang, Jia-Shun Wu, Hong-Chun Xian, Bing-Jun Chen, Hao-Fan Wang, Xiang-Hua Yu, Xin Pang, Li Dai, Jian Jiang, Xin-Hua Liang, Ya-Ling Tang

**Affiliations:** 1 State Key Laboratory of Oral Diseases & National Clinical Research Center for Oral Diseases, West China Hospital of Stomatology (Sichuan University), Chengdu 610041, China; 2 Department of Head and Neck Surgery, Sichuan Cancer Hospital, Sichuan Cancer Center, School of Medicine, University of Electronic Science and Technology of China, Chengdu, Sichuan, China

**Keywords:** salivary adenoid cystic carcinoma (SACC), CXCR5, perineural invasion (PNI), microRNAs (miRNAs), Schwann cells

## Abstract

CXCR5 played critical roles in tumorigenesis and metastasis. Nevertheless, little was known about the involvement of CXCR5 in perineural invasion (PNI) of salivary adenoid cystic carcinoma (SACC). Here, we confirmed upregulation of CXCR5 in SACC specimens and cells and identified that CXCR5 exhibited a significant positive correlation with PNI. Functionally, knockdown of CXCR5 suppressed SACC cells migration, invasion and PNI ability, whereas CXCR5 overexpression displayed the opposite effects. Moreover, CXCR5 downregulated microRNA (miR)-187, which could competitively sponge S100A4. The PNI-inhibitory effect of CXCR5 knockdown or miR-187 overexpression could be reversed by elevated expression of S100A4. Conjointly, our data revealed that CXCR5 facilitated PNI through downregulating miR-187 to disinhibit S100A4 expression in SACC.

## INTRODUCTION

Salivary adenoid cystic carcinoma (SACC) belongs to malignant epithelial tumor derived from the salivary gland, accounting for about 28% of salivary gland malignancies [1–3]. Despite the progress in surgical resection combination with radiotherapy and/or chemotherapy for SACC, more than 30% of the SACC patients still experience local recurrence and hematogenous spread to distant organs after primary treatments [4–7]. One of the pivotal factors affecting local recurrence or distant metastasis of SACC is perineural invasion (PNI), which refers to tumor cells encroaching along nerves. PNI can hinder curative resection and is predicted to reduce survival rate [8–10]. Thus, it is necessary to elucidate the concrete mechanism driving PNI, which may guide us with a better management of SACC.

The chemokine receptor CXCR5, pertaining to G-protein coupled receptors (GPCR) superfamily, could promote lymphocyte migration and evoke inflammatory responses [11, 12]. Increasing evidence revealed that CXCR5 was highly expressed in many human cancers and was related to occurrence, invasion and metastasis of tumors [13–15]. Recent studies reported that CXCR5 was overexpressed and associated with PNI in colorectal and prostate cancers [16, 17]. Therefore, the role of CXCR5 in PNI of SACC is a burgeoning field of research.

MicroRNAs (miRNAs) are a class of single-stranded non-coding RNAs with a length of 18-25 nucleotides. MiRNAs work via forming RNA-induced silencing complex (RISC) to inhibit mRNAs translation, and they also trigger degradation of target mRNAs through binding to the 3′-UTRs [18, 19]. Moreover, miRNAs emerging as robust players of PNI in perineural niche have aroused wide interest [20]. However, whether miRNAs participate in CXCR5-driven PNI of SACC has not been characterized.

To invade the nerve, tumor cells must rearrange their skeletons and change their morphology to obtain stronger invasiveness. Schwann cells, which have been verified as the main cells of peripheral nerves, are fundamental to the survival and development of nerves and conducive to the maintenance of axons [21, 22]. Substantial evidences suggested that the expression of Schwann cell hallmarks, including S100 calcium binding protein A4 (S100A4), p75 neurotrophin receptor (p75NTR) and glial fibrillary acidic protein (GFAP) were increased considerably in many tumors with PNI, such as melanoma [23], breast cancer [24], prostate cancer [25], colorectal cancer [26]. Therefore, scholars speculated that the differentiation of tumor cells into Schwann-like cells could increase migration and invasion ability of tumor cells. And the Schwann-like differentiation might be one of the mechanisms of PNI occurrence in tumors [27, 28]. In the SACC, Shan et al. found that the expression of S100A4 and GFAP at nerve invasion frontier was dramatically increased [29]. Thereby, we focused on whether CXCR5 could initiate a miRNA mediated network to drive the differentiation of tumor cells into Schwann-like cells in PNI of SACC, promising to identify new biomarkers and offer cancer therapeutic targets.

## MATERIALS AND METHODS

### Patients and specimen collection

158 patients suffering from SACC and receiving curative surgical resection without chemotherapy, radiotherapy or hormone therapy prior to surgery at West China Hospital of Stomatology, Sichuan University between 2002 and 2007 were enrolled in the cohort with informed consent. The clinicopathological TNM stages were determined according to the International Union Against Cancer TNM classification of malignant tumors [30]. And 20 cases of normal salivary glands adjacent to carcinoma were randomly selected as control. The follow-up information was up-dated on December 31, 2017. The median follow-up of these patients was 83.7 months (range 3.5–140 months). The present study protocol was approved by the Institutional Ethics Committee of West China Medical Center, Sichuan University, China.

### Immunohistochemistry (IHC)

All samples were fixed in formaldehyde solution, embedded in paraffin and sectioned for immunohistochemistry (IHC) analysis according to IHC staining procedure. Primary antibodies for CXCR5 (1:250), S100A4 (1:100), p75NTR (1:100), GFAP (1:200) were purchased from Abcam (Cambridge, MA). The slides were observed using DAB and counterstained with hematoxylin. The stained slides were evaluated by two pathologists independently without the knowledge of patients’ clinicopathological parameters. The immunoreactive intensity was assigned to − (0), <5%; + (1), 5-25%; ++ (2), 25-50%; +++ (3), >50%, representing negative expression, weakly positive expression, moderately positive expression and strongly positive expression, respectively. − and + mean low expression, and ++ and +++ mean high expression.

### Cell line and cell culture

SACC cell lines (SACC-83 and SACC-LM) and nerve cell line microglia BV2 were provided by State Key Laboratory of Oral Diseases & National Clinical Research Center for Oral Diseases, West China Hospital of Stomatology, Sichuan University and maintained in Dulbecco’s Modified Eagle’s Medium (DMEM) (Gibco, US), supplemented with 10% fetal bovine serum (FBS) (Gibco, US), 50 mg/ml streptomycin and 50 unit/ml penicillin (Gibco, US) at 37°C with 5% CO2 in humidified incubators.

### Cell transfection

SiRNA targeting CXCR5, negative control siRNA (Control), negative control miRNA mimic (NC mimic), miR-187 mimic (miR-187), negative control miRNA inhibitor (NC inhibitor) and miR-187 inhibitor were synthesized by GenePharma. CXCR5 and S100A4 were integrated into pcDNA3.1 (Invitrogen, Carlsbad, CA) for overexpression experiments. SACC cells at ~50% confluence were transfected with Lipofectamine 2000 reagent (Invitrogen, Carlsbad, CA) in conformity with the manufacturer’s protocol. Besides, blank group (only transfection reagent) was used as blank control group (Blank). The medium containing Lipofectamine 2000 was changed into RPMI-1640 with 10% FBS after 6 h. The cells were collected for the following experiments after 48 h transfection.

### Immunofluorescence staining

SACC cells were seeded on coverslips, incubated for 24 h, fixed with 4% formaldehyde for 15 min, permeabilized with 0.5% Triton X-100 for 10 min and blocked with 10% normal goat serum for 1 h. Then, primary antibodies against CXCR5 were incubated overnight at 4°C. Next day, cells were incubated with fluorescein-conjugated secondary antibodies for 1 h and the nuclei was stained with 4′, 6-diamidino-2-phenylindole (DAPI). Images were captured using a fluorescence microscope (Leica, Germany).

### Quantitative real-time PCR (qRT-PCR)

Total RNA was extracted from cells and tissue using TRIzol reagent (Takara, Tokyo, Japan) and the quantity and quality of RNA were measured with the NanoDrop ND-1000 Spectrophotometer (Thermo Scientific Inc., Waltham, MA). Then, RNA was reverse-transcribed into miRNA cDNA and total cDNA respectively using One Step PrimeScript miRNA cDNA Synthesis Kit and SuperScript™ III First-Strand Synthesis Kit, respectively. QRT-PCR was conducted according to the protocol provided by the manufacturer using the PCR primer sequences in Supplementary Table 1. U6 was served as an endogenous control for miRNA, and mRNA were normalized to GAPDH. Each experiment was conducted in triplicate.

### Scratch wound healing assay

1.0 μg/ml of anti-human CXCR5 antibody (R&D Systems) was added to 6-well plates after SACC cells were seeded [17]. When SACC cells were at a density of 95%, scratches were created with a 200 μL pipette tip and detached cells were removed with PBS rinsing. And the scratches were observed and photographed at 0 h and 24 h. Percentage of wound closure was statistically analyzed using Image-Pro Plus Analysis software (Media Cybernetics company, Rockville, Maryland).

### Transwell invasion assay and co-culture assay

For the Matrigel invasion assays, SACC cells (1×10^5^) were seeded into the upper chamber of the Corning Matrigel Invasion insert covered with Matrigel (pore size, 8.0 μm; BD Biosciences) containing 200 μL serum-free medium with 1.0 μg/ml of anti-human CXCR5 antibody. The bottom chamber was filled with 500 μL medium containing 10% FBS. The cells were fixed in 4% paraformaldehyde and then stained with 0.05% crystal violet after 48 h. Cells penetrated the membrane to the bottom of the chamber were counted in five random fields.

To investigate the role of CXCR5 in PNI of SACC, co-culture assay was conducted based on the model previously employed by He et al. [31, 32]. In brief, BV2 cells (1×10^5^) were seeded into the 24-well plates. The next day, Boyden Chamber was inserted and SACC cells were seeded into Boyden Chamber covered with Matrigel and treated with 1.0 μg/ml of anti-human CXCR5 antibody. The number of invasive cells penetrating the membrane to the bottom of the chamber was counted after Transwell chamber was incubated for 48 h.

### TRITC-phalloidin staining for detecting cytoskeletal reorganization

SACC cells were fixed with 4% paraformaldehyde for 15 min, lysed with 0.2% Triton X-100 for 15 min and blocked with 5% BSA for 20 min. Immediately, cytoskeleton dye TRITC-phalloidin (Sigma) was incubated for 30 min and DAPI was used for nuclei staining. Images were visualized and captured using a fluorescence microscope.

### MiRNA microarray

Total RNA of control or si-CXCR5 SACC-LM cells was extracted with the TRIzol reagent (Invitrogen), purified using the NanoPhotometer^®^ spectrophotometer (IMPLEN, CA) and subjected to a miRNA microarray. Data analysis was performed via GeneSpring software (Agilent). For selecting differently expressed genes, absolute value fold change of at least 1.5 and a *p*-value less than 0.05 was used as the cut-off value.

### Fluorescent *in situ* Hybridization (FISH)

Fish analysis was conducted on paraffin section. Cy3-labeled miR-187 and FISH Kit (RiboBio, Guangzhou, China) were used following the manufacturer's instructions.

### Luciferase reporter assay

Wild-type (Wt) and mutated-type (Mut) human S100A4 3′-UTR containing the miR-187 binding site were synthesized and co-transfected with miR-187 mimic or NC mimic into SACC cells. After 48 h of transfection, luciferase activity was determined by Dual-Luciferase Reporter Assay System and normalized to Renilla luciferase in each well.

### *In vivo* PNI model

To determine whether CXCR5 played an essential role in the occurrence of PNI in SACC, 16 athymic nude mice aged 5 weeks were purchased. Under sterile conditions, the mice received groin injection of 2.5×10^6^ SACC-LM cells in phosphate buffered saline (PBS). 2 weeks after inoculation, 16 nude mice were randomly allocated into two group, including saline group (*n* = 8, treatment with saline at 0.1 ml/day) and anti-CXCR5 antibody group (*n* = 8, treatment with anti-CXCR5 antibody at 1.0 μg/day injected into the tumor site), which referenced and improved previous study about the usage of antibody *in vivo* [33, 34], and receive corresponding treatment for 2 weeks, respectively. Sciatic nerve function, which intervened functional movement of hind limbs in nude mice was monitored for 2 weeks. Tumor was surgically removed and subsequent experiments were conducted.

### Statistical analysis

The expression of CXCR5 in different PNI status and the association between CXCR5 expression and patients’ clinicopathologic parameters were analyzed by Wilcoxon test. The relationship between CXCR5 expression and the expression of Schwann cell hallmarks at nerve invasion front was evaluated by Spearman's rank correlation analysis. SPSS software package 21.0 (SPSS, Chicago, IL, USA) was used to conduct the statistical analyses. Differences were considered statistically significant when *p* value was less than 0.05 and statistical differences among multiple groups were corrected using Bonferroni for multiple testing.

### Ethics approval and consent to participate

The use of human tissue samples and clinical data was approved by the Institutional Ethics Committee of the West China Medical Center, Sichuan University, China (WCHSIRB-D-2016-207, WCHSIRB-D-2017-120). The written informed consents were obtained from participants through their signatures. All procedures involving animal were approved by the Subcommittee on Research and Animal Care (SRAC) of Sichuan University (WCHSIRB-D-2016-191).

### Availability of data and material

All data generated or analyzed during this study are included in this published article and its supplementary files.

## RESULTS

### Overexpression of CXCR5 contributes to PNI in SACC samples

**Figure 1:**
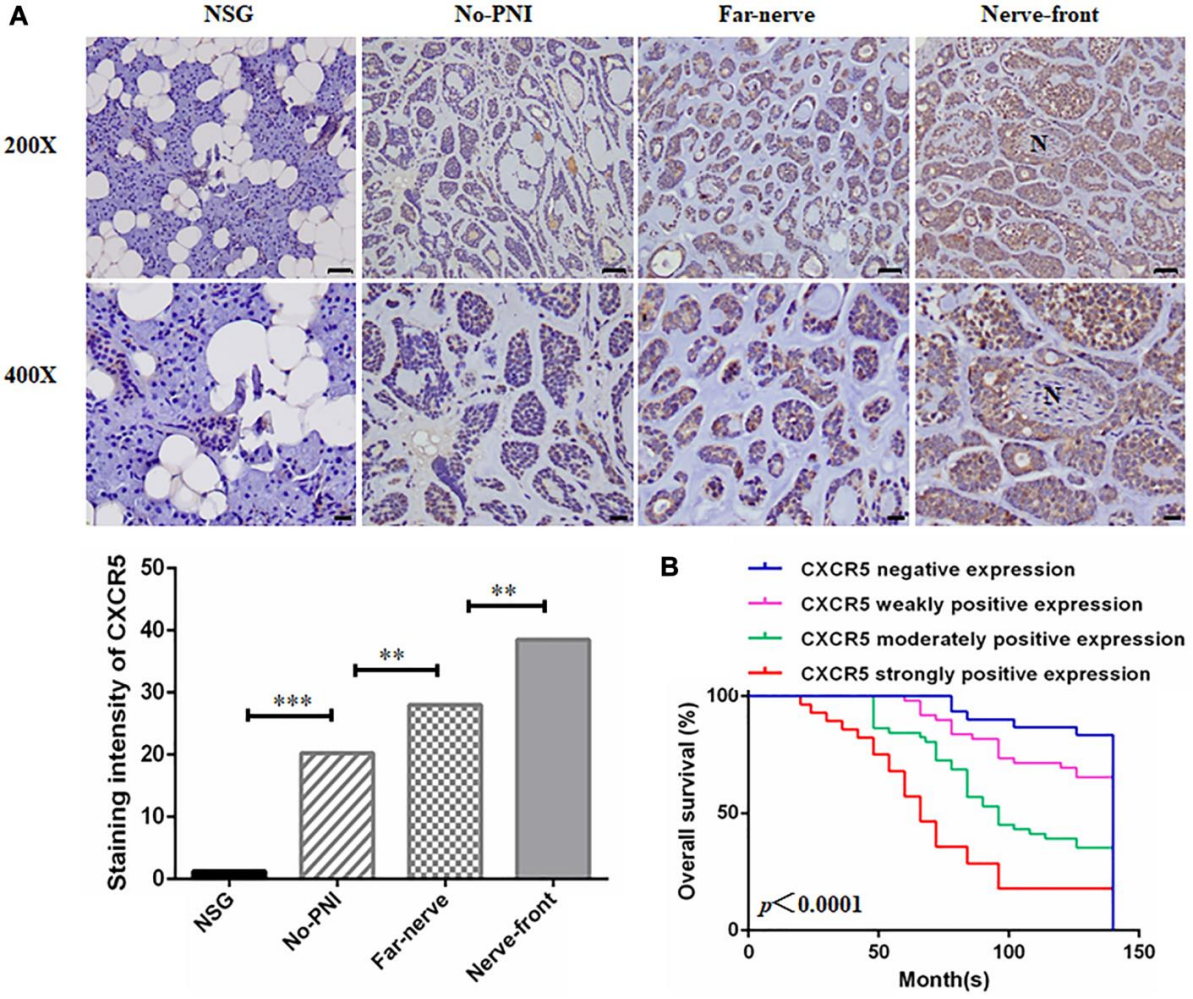
**Clinical significance of CXCR5 expression in SACC patients.** (**A**) Immunohistochemical staining of CXCR5 in normal salivary gland (NSG), SACC without PNI (No-PNI), far away from nerve of SACC with PNI (Far-nerve), nerve invasion front of SACC with PNI (Nerve-front). ‘N’ represented nerve. (Bar: upper, 50 μm; lower, 20 μm) (^**^p < 0.01, ^***^p < 0.001). (**B**) Kaplan-Meier survival analysis on SACC patients with different expression status of CXCR5. The overall survival time for patients without or with low CXCR5 expression was longer than those with high CXCR5 expression (log-rank test, *p* < 0.0001).

Among 158 SACC cases, the incidence of PNI is 48.7% (77/158) via HE staining. The clinicopathologic characteristics of patients were summarized in Table 1. IHC staining showed that CXCR5 was generally expressed in cytoplasm and/or cytomembrane, with 75% (15/20) negative expression and 25% (5/20) weak expression out of 20 cases normal salivary gland (NSG). However, the positive rate of CXCR5 was 81.0% (128/158) in SACC specimens. The expression of CXCR5 in SACC specimens was noticeably higher versus NSG group (*p* < 0.0001) (Figure 1A). CXCR5 expression was significantly associated with patients’ tumor site, clinical stage, involvement of surgical margin, local regional recurrence, distant metastasis and histological subtype (*p* = 0.038, *p* = 0.017, *p* = 0.04, *p* = 0.008, *p* = 0.001 and *p* = 0.008, respectively), but not with age, sex, complaint and tumor size (all *p >* 0.05) (Table 1). Subsequently, Kaplan-Meier curve revealed that SACC patients with high CXCR5 expression level had a significantly worse overall survival (OS) than those with lower CXCR5 levels (Figure 1B). Multivariate Cox Regression analysis indicated that the elevated CXCR5 level was an independent prognosticator of OS for SACC patients (*p* = 0.003) (Table 2).

**Table 1:** Relation of CXCR5 expression with clinicopathological parameters in 158 cases of SACC.

**Clinicopathological parameters**		**cases**	**CXCR5 expression**				***p* value**
			**−**	**+**	**++**	**+++**	
		158	30	49	51	28	
Ages (years)	<50	73	12	27	23	11	0.642
	≥50	85	18	22	28	17	
Sex	Male	85	20	26	27	12	0.104
	Female	73	10	23	24	16	
Complaint (months)	<12	83	16	31	23	13	0.202
	≥12	75	14	18	28	15	
Site	Minor salivary gland	108	19	27	41	21	0.038
	Major salivary gland	50	11	22	10	7	
Tumor diameter (cm)	≤1	24	8	5	7	4	0.612
	1~2	45	9	13	13	10	
	≥2	89	13	31	31	14	
Clinical stage	I+II	70	24	13	24	9	0.017
	III+IV	88	6	36	27	19	
Histological subtype	Cribriform	72	13	28	27	4	0.008
	Tubular	49	12	12	18	7	
	Solid	37	5	9	6	17	
Involvement of surgical margin	Affect	48	6	12	19	11	0.04
	free	110	24	37	32	17	
Local regional recurrence	Positive	41	5	11	10	15	0.008
	Negative	117	25	38	41	13	
Distant metastasis	Positive	46	7	6	19	14	0.001
	Negative	112	23	43	32	14	

**Table 2:** Cox multivariate regression analysis of overall survival in SACC patients.

	**B**	**SE**	**Wald**	**df**	**Sig.**	**Exp (B)**	**95.0% CI for Exp (B)**	
							**Lower**	**Upper**
Clinical stage	0.749	0.280	7.140	1	0.008	2.115	1.221	3.663
Involvement of surgical margin	0.384	0.262	2.161	1	0.142	1.469	0.880	2.452
Local regional recurrence	0.892	0.292	9.357	1	0.002	2.440	1.378	4.320
PNI	0.728	0.254	8.212	1	0.004	2.070	1.259	3.405
Distant metastasis	0.884	0.254	12.085	1	0.001	2.421	1.471	3.985
CXCR5 expression	0.407	0.135	9.027	1	0.003	1.502	1.152	1.959

To investigate the role of CXCR5 in the PNI of SACC, CXCR5 expression in different PNI status was assessed, and the result revealed that the expression of CXCR5 was increased in the samples with PNI compared to the no-PNI group (*p* = 0.005). And CXCR5 expression levels displayed an increasing trend from far away from nerve site (Far-nerve group) to nerve invasion frontier (Nerve-front group) in SACC with PNI (*p* = 0.001) (Table 3) (Figure 1A). These data suggested that the occurrence of SACC was correlated to the expression of CXCR5, and the high CXCR5 expression was found to be associated significantly with the presence of PNI in SACC.

**Table 3:** The expression of CXCR5 in normal salivary gland and SACC with different PNI status.

	**Cases**	**NSG**		**No-PNI**		**Far-nerve**		**Nerve-front**	***p* value**
		**(*n* = 20)**		**(*n* = 81)**		**(*n* = 77)**		**(*n* = 77)**	
CXCR5	−	15		26		16		4	<0.0001
	+	5		34		25		15	
	++	0		16		21		35	
	+++	0		5		15		23	
	*p* value		0.0001		0.005		0.001		

NOTE: NSG, normal salivary gland group.

No-PNI, SACC without PNI group.

Far-nerve, far away from nerve of SACC with PNI group.

Nerve-front, nerve invasion front of SACC with PNI group.

### CXCR5 promotes migration, invasion and PNI in SACC cells

**Figure 2:**
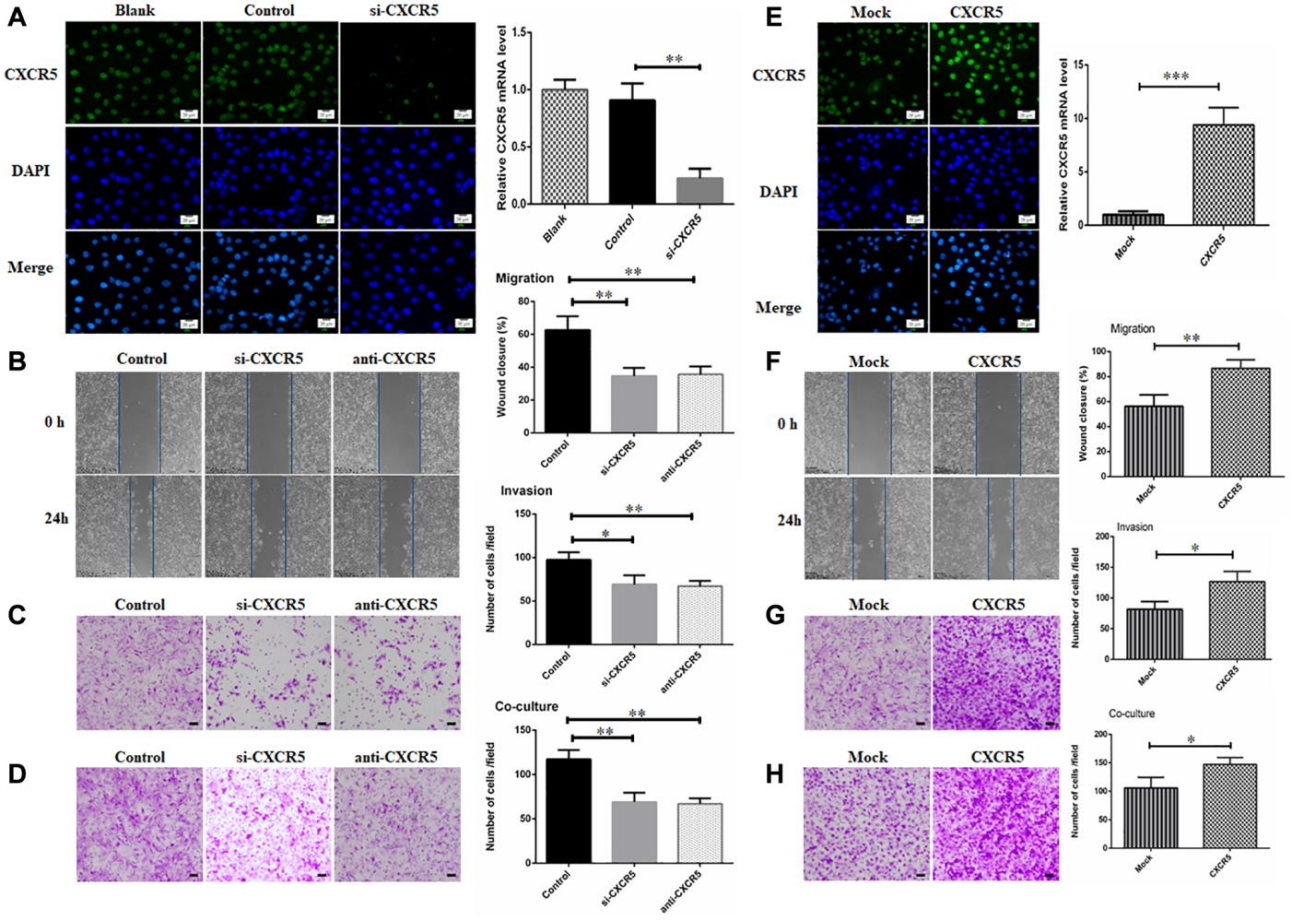
**Silencing CXCR5 attenuates migration, invasion and PNI in SACC cells.** (**A**) Immunofluorescence and qRT-PCR analysis showed that CXCR5 expression was dramatically inhibited by siRNA treatment. (**B**–**D**) CXCR5 silence with siRNA or CXCR5 blockade with anti-CXCR5 antibody impeded migration, invasion and PNI of SACC-LM cells, (Bar: 100 μm). (**E**) Immunofluorescence and qRT-PCR analysis showed that CXCR5 level was significantly increased by CXCR5-overexpressing plasmid. (**F**–**H**) CXCR5 overexpression enhanced migration, invasion and PNI capacity of SACC-LM cells, (Bar: 100 μm) (^*^p < 0.05, ^**^p < 0.01, ^***^p < 0.001).

To further ascertain the relationship of CXCR5 and PNI in SACC, siRNA-mediated knockdown assays were performed. CXCR5 level was significantly inhibited after siRNA treatment in SACC cells by immunofluorescence and qRT-PCR analysis, and representative results in SACC-LM cells were displayed in Figure 2A. We observed that CXCR5 knockdown in SACC cells impeded migration in wound healing assays (Figure 2B) and invasion in Matrigel invasion assays (Figure 2C). Meanwhile, the invasive potential of SACC cells towards nerve cell BV2 was also weakened after CXCR5 silencing (Figure 2D). Similarly, CXCR5 blockage induced by anti-CXCR5 antibody notably inhibited migration, invasion and PNI of SACC cells. Moreover, CXCR5-overexpressing plasmid was transfected in SACC-LM cells (Figure 2E), which markedly contributed to migration, invasion and PNI of SACC-LM cells compared with mock cells (Figure 2F–2H).

### Schwann-like cell differentiation is involved in CXCR5 induced PNI in SACC cells

**Figure 3:**
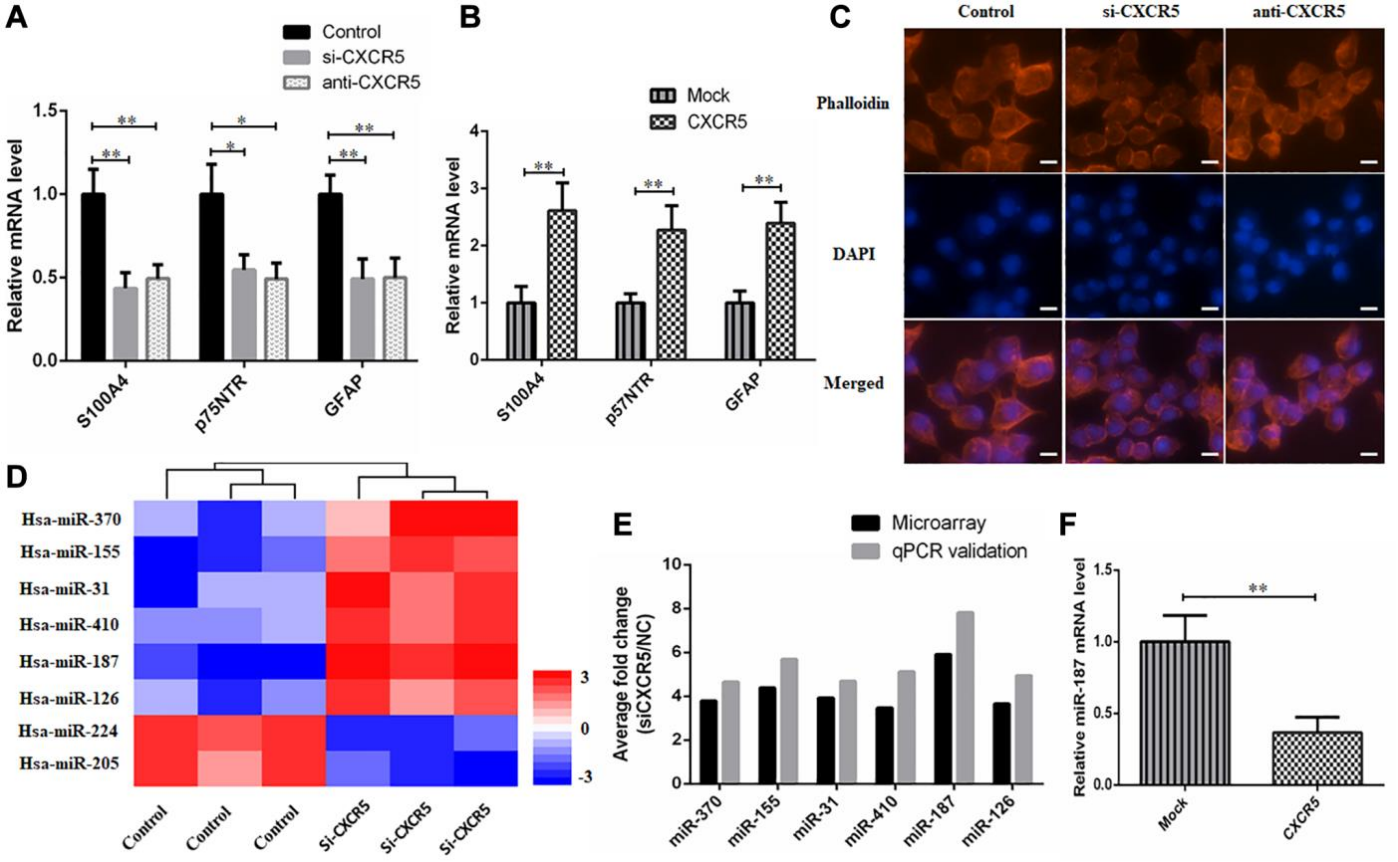
**Schwann-like cell differentiation is involved in CXCR5 induced PNI in SACC cells.** (**A**) CXCR5 silence with siRNA or CXCR5 blockade with anti-CXCR5 antibody downregulated the expression of Schwann cell markers, including S100A4, p75NTR, GFAP by qRT-PCR analyses. (**B**) CXCR5 overexpression upregulated the expression of S100A4, p75NTR, GFAP in SACC-LM cells. (**C**) CXCR5 silence with siRNA or CXCR5 blockade with anti-CXCR5 antibody resulted in the transformation of SACC-LM cells from spindle-like fibroblastic cellular morphology to epithelial plasticity with little pseudopodia, (Bar: 50 μm). (**D**) The heatmap of 8 differentially expressed miRNAs in SACC-LM cells transfected with control or si-CXCR5 on a scale from blue (low) to red (high). ‘Blue’ represents low expression, and ‘red’ represents high expression. (**E**) The microarray data of 8 differentially expressed miRNAs in the si-CXCR5 and control SACC-LM cells was confirmed by qRT-PCR. For qRT-PCR, U6 was used to normalize the Ct values. (**F**) CXCR5 overexpression upregulated the expression of miR-187 in SACC-LM cells, (^*^p < 0.05, ^**^p < 0.01).

Schwann-like cell differentiation promoted migration and invasion of tumor cells [29]. Hence, we analyzed hallmarks of Schwann cell in CXCR5-induced SACC cells during PNI. Here, we found that knockdown or blockade of CXCR5 brought about a considerable downregulation of Schwann cell hallmarks, including S100A4, p75NTR, GFAP in SACC-LM cells compared with the control group (Figure 3A), whereas overexpression of CXCR5 induced upregulation of S100A4, p75NTR, GFAP (Figure 3B). Moreover, SACC cells underwent the transformation from an elongated, invasive, spindle-like fibroblastic cellular morphology to epithelial plasticity with cobblestone-like appearance and little branching after CXCR5 silence or blockade in SACC-LM cells (Figure 3C).

### CXCR5 promotes the expression of Schwann cell markers by inhibiting miR-187

To determine the downstream miRNAs of CXCR5, SACC-LM cells were transfected with control siRNA or si-CXCR5 and microarray was performed to screen differentially expressed miRNAs. 8 differentially expressed miRNAs showed in Figure 3D were selected to validate by qPCR (Figure 3E) and miR-187 was the one with most dynamic expression. Based on the result, we investigated the effect of CXCR5 overexpressing on miR-187 and observed a remarkable downregulated trend of miR-187 (Figure 3F), thus we selected miR-187 for further investigation.

**Figure 4:**
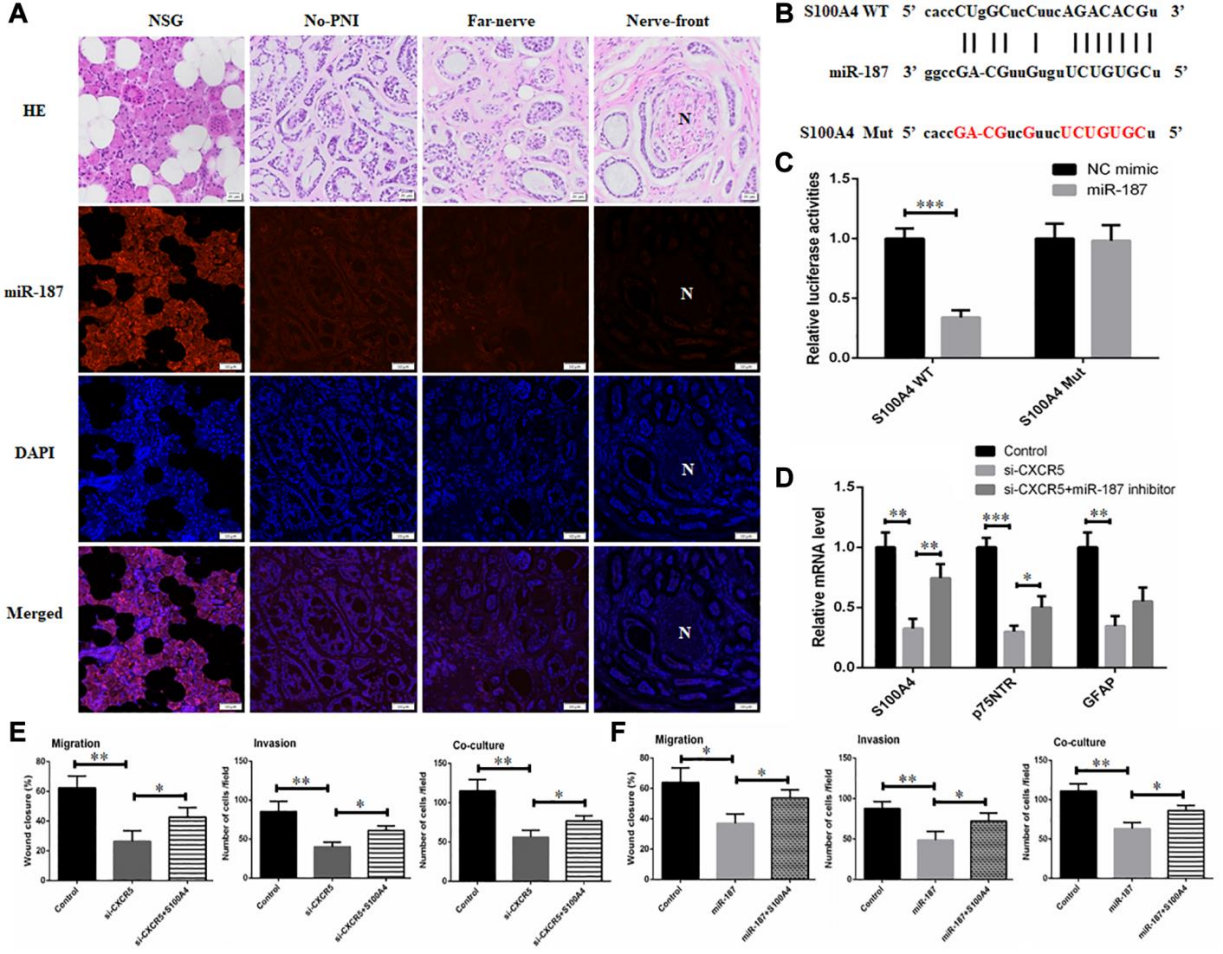
**CXCR5 promotes the expression of Schwann cell markers by inhibiting miR-187.** (**A**) An RNA-FISH assay was conducted to determine the level of miR-187 in normal salivary gland (NSG), SACC without PNI (No-PNI), far away from nerve of SACC with PNI (Far-nerve), nerve invasion front of SACC with PNI (Nerve-front). ‘N’ represented nerve, (Bar: 50 μm). (**B**) The binding sites on S100A4 3′UTR for miR-187 and the mutant sites. (**C**) Luciferase reporter analysis was carried out to determine the interaction of miR-187 with S100A4. (**D**) qRT-PCR analysis showed that reduced expression of Schwann cell hallmarks mediated by CXCR5 silence could be partially reversed by miR-187 inhibitor. (**E**) Inhibitory migratory, invasive and PNI ability in SACC-LM cells mediated by CXCR5 silence could be reversed by S100A4 overexpression. (**F**) Repressive migratory, invasive and PNI ability in SACC-LM cells induced miR-187 overexpression was alleviated by S100A4 overexpression, (^*^p < 0.05, ^**^p < 0.01).

Next, we conducted RNA FISH assay using paraffin-embedded tissue samples, and results validated the downregulation of miR-187 in SACC tissues compared with NSG. In addition, miR-187 level presented a downward trend at nerve invasion frontier (Figure 4A). Thereafter, we predicted whether miR-187 had the potential to interact with Schwann cell markers via the bioinformatic tool starBase, and identified a putative binding site for miR-187 in the S100A4 sequence. Thus, we reasoned that CXCR5 manipulated PNI of SACC through repressing miR-187 to disinhibit S100A4. The binding sites on miR-187 and S100A4 3′UTR were presented in Figure 4B. Luciferase reporter assay showed that overexpressing miR-187 provoked efficient quenching the luciferase activity in S100A4 WT, but not notable difference in the S100A4 Mut (Figure 4C). QPCR analysis showed that the reduction of Schwann cell hallmarks mediated by CXCR5 silence could be partially relieved by miR-187 inhibitor (Figure 4D).

Those findings supported that miR-187 targeted and inhibited S100A4 activity in a direct binding manner. Further, inhibitory migratory, invasive and PNI ability in SACC-LM cells mediated by CXCR5 silence or miR-187 overexpression could be reversed by S100A4 overexpression (Figure 4E–4F).

### Inhibition of CXCR5 suppresses PNI in SACC xenograft model

**Figure 5:**
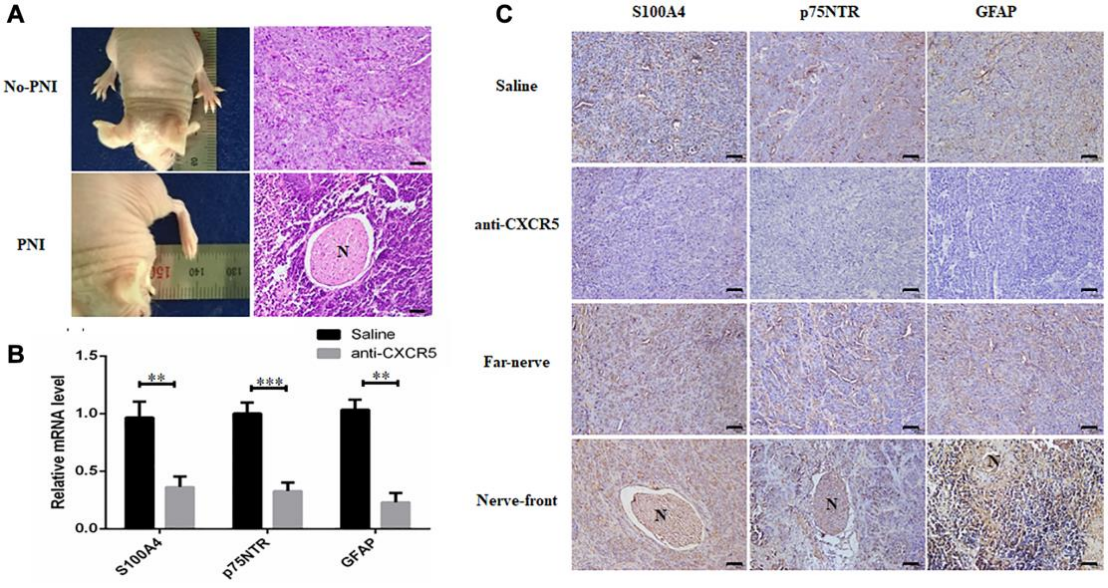
**Inhibition of CXCR5 suppresses PNI in SACC xenograft model.** (**A**) Representative images on hindlimb function of the mouse and HE staining were presented. (**B**) qRT-PCR analysis showed that anti-CXCR5 antibody group mice showed reduced expression of S100A4, p75NTR and GFAP compared with saline group mice. (**C**) Immunohistochemical staining showed that anti-CXCR5 antibody group mice showed reduced expression of S100A4, p75NTR and GFAP compared with saline group mice. The expression of S100A4, p75NTR, GFAP was higher at nerve invasion frontier than that in tumors far away from nerve in PNI groups, ‘N’ represented nerve, (Bar: 50 μm) (^**^p < 0.01, ^***^p < 0.001).

To further validate our findings, *in vivo* PNI model was conducted as described in methods. The results displayed that compared with saline group, the mice in anti-CXCR5 antibody group underwent less contracture and dysfunction of hindlimb paw, which was intervened by sciatic nerve. Xenografted tumors were excised for HE staining, and the results showed the incidence of PNI in saline group and anti-CXCR5 antibody group was 62.5% (5/8) and 12.5% (1/8), respectively (Figure 5A). The incidence of PNI was lower in anti-CXCR5 antibody group compared with saline group. Besides, the results of qRT-PCR (Figure 5B) and immunohistochemistry analysis (Figure 5C) showed that the expression of S100A4, p75NTR and GFAP was higher in anti-CXCR5 antibody group than that in saline group, besides, it was significantly increased at nerve invasion frontier in PNI group compared with tumors far away from nerve.

### CXCR5 expression intrinsically connects with the level of Schwann cell markers in SACC specimens

**Figure 6:**
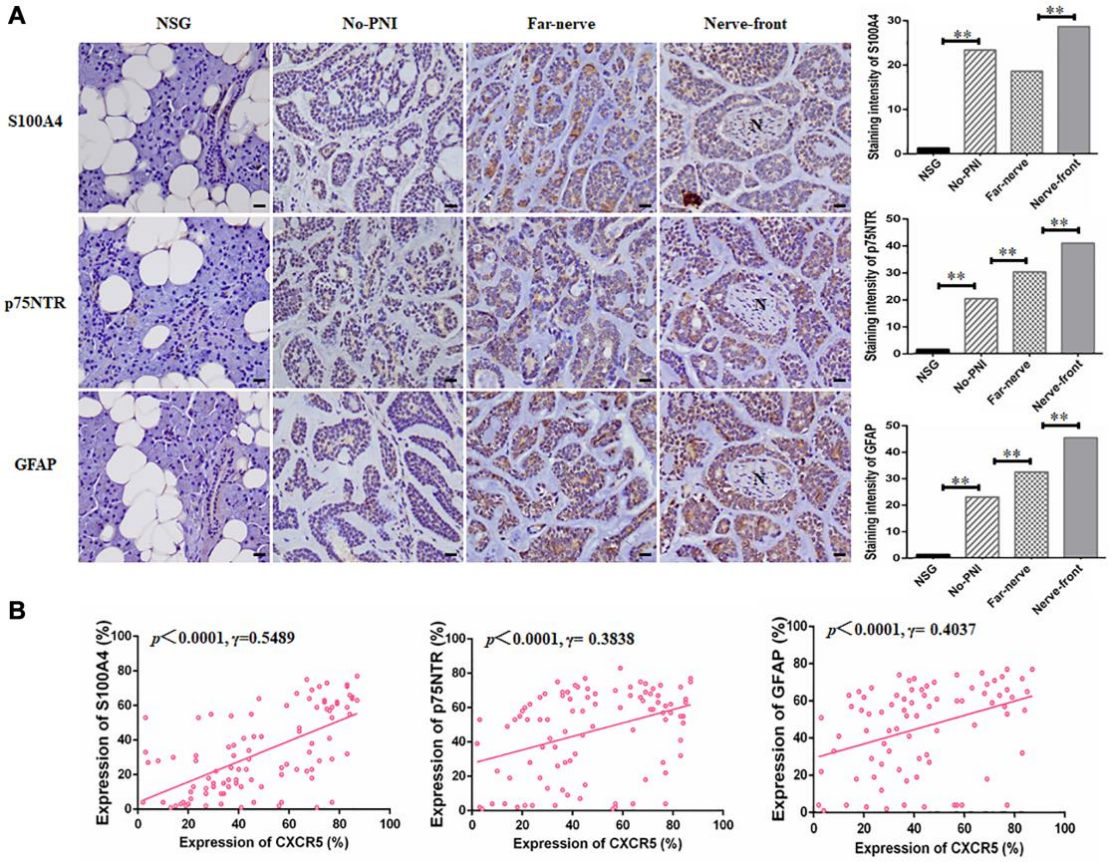
**CXCR5 expression intrinsically connects with the level of Schwann cell markers in SACC specimens.** (**A**) Immunohistochemical staining of Schwann cell markers, including S100A4, p75NTR and GFAP in normal salivary gland (NSG), SACC without PNI (No-PNI), far away nerve of SACC with PNI (Far-nerve), nerve invasion front of SACC with PNI (Nerve-front). ‘N’ represented nerve, (Bar: 20 μm). (**B**) Correlation of CXCR5 with Schwann cell markers at nerve invasion frontier of SACC patients. CXCR5 expression was positively correlated with the expression of S100A4, p75NTR and GFAP at nerve invasion frontier of SACC (^**^p < 0.01).

Schwann cell related markers, including S100A4, p75NTR, GFAP were also examined by immunohistochemistry staining in 158 SACC specimens and 20 normal salivary glands (Figure 6A, Supplementary Figure 1). The associations between CXCR5 and Schwann cell hallmarks, including S100A4, p75NTR, GFAP at nerve invasion frontier of PNI groups in SACC were assessed. The results showed that CXCR5 expression was positively correlated with the expression of S100A4 (*p* < 0.0001, γ = 0.5489), p75NTR (*p* < 0.0001, γ = 0.3838) and GFAP (*p* < 0.0001, γ = 0.4037) (Figure 6B).

## DISCUSSION

PNI not only brings about dysfunction, but also causes the recurrence and metastasis in multiple malignant neoplasm, including head and neck [3, 9], pancreatic [35], prostate [10], colorectal [36], gastric [37], breast [8] cancer, and so on. In the present study, we found that CXCR5 was highly expressed in SACC tissue compared with normal salivary gland, and CXCR5 expression was significantly correlated with tumor site, clinical stage, histological subtype, involvement of surgical margin, local regional recurrence and distant metastasis. To our knowledge, this is the first study to confirm the possible clinical relevance of CXCR5 to SACC patients. To explore the relationship between CXCR5 and PNI, CXCR5 expression in different PNI status was also analyzed, and the results showed that CXCR5 expression at nerve invasion frontier was higher than far away from nerve site in SACC specimens. To verify above findings, we extended our research *in vitro*. The results showed that the migration and invasion and PNI of SACC cells could be inhibited by CXCR5 silence with siRNA or CXCR5 blockade with anti-CXCR5 antibody.

And these results were in accordance with the previous studies, which revealed that CXCR5 participated in the progression and poor prognosis in many types of cancers. For example, Li et al. confirmed that CXCR5 was significantly elevated and associated with the poor prognosis of HCC patients [38]. Singh et al. noted that CXCR5 accelerated malignant progression of lung cancer, and inhibition of CXCR5 may provide a new treatment strategy for lung cancer [39]. Rubenstein et al. identified that CXCR5 was promised to be a new target for the detection and treatment of lymphoma [40]. Qi et al. demonstrated that CXCR5 played a pivotal role in the growth, migration and invasion of colon cancer cell through PI3K/AKT pathway [16]. Singh et al. verified that CXCR5 mediated prostate cancer cell proliferation and invasion through the activation of Src, FAK, JNK and ERK1/2 signaling [17, 41]. Biswas et al. reported that CXCR5 induced invasion and metastasis through the activation of Src kinase signaling pathway in breast cancer [42].

Enhanced Schwann-like cell differentiation companied with cytoskeleton rearrangement and the pseudopodia formation of tumor cells is a crucial prerequisite for PNI [29, 43–45], by which tumor cells acquire fibroblast like morphology and mesenchymal traits to invade and metastasize. In this study, the elevated CXCR5 level was constantly accompanied by upregulated expression of S100A4, p75NTR and GFAP, which had been characterized as Schwann cell hallmarks [21, 46] at nerve invasion frontier. Decreased CXCR5 inhibited the expression of S100A4, p75NTR and GFAP and promoted transformation of SACC cells from spindle-like fibroblastic cellular morphology to epithelial plasticity with little pseudopodia. These revealed that Schwann-like cell differentiation might occur and CXCR5 might involve in the process of Schwann-like cell differentiation in PNI of SACC cells.

Dysregulation of miRNAs, which negatively orchestrate the expression of their target gene by binding to complementary 3′-UTR sequences of target mRNA, is known as an important mechanism contributing to PNI of tumors. In our present study, to further explore the role of miRNA in CXCR5 induced PNI of SACC, miRNA array analysis and potential target relationship prediction by Targetscan were performed. We found that miR-187, which harbored S100A4 binding sites, was the most increased miRNA after CXCR5 loss. Furthermore, we illustrated that miR-187 was markedly downregulated at the nerve invasion frontier by FISH. Dual luciferase reporter assays and rescue experiments confirmed CXCR5-dependent regulation of PNI was achieved by miR-187 silence to derepress S100A4 expression. The inhibitory effect of migration, invasion and PNI by CXCR5 knockdown or miR-187 overexpression could be reversed through S100A4 overexpression.

Taken together, the present study demonstrated that CXCR5 triggered the differentiation of tumor cells into Schwann-like cells through repressing miR-187 to disinhibit S100A4, thus facilitating PNI of SACC.

## Supplementary Materials

Supplementary Figure 1

Supplementary Table 1
