# Supplementary material for: CXCR5 induces perineural invasion of salivary adenoid cystic carcinoma by inhibiting microRNA-187
Source: Aging (Albany NY). 2021 Jun 10;13(11):15384–99. doi: 10.18632/aging.203097 (PMC8221347; doi:10.18632/aging.203097)
Supplement: Supplementary Figure 1 [file aging-13-203097-s001.pdf]

SUPPLEMENTARY FIGURE

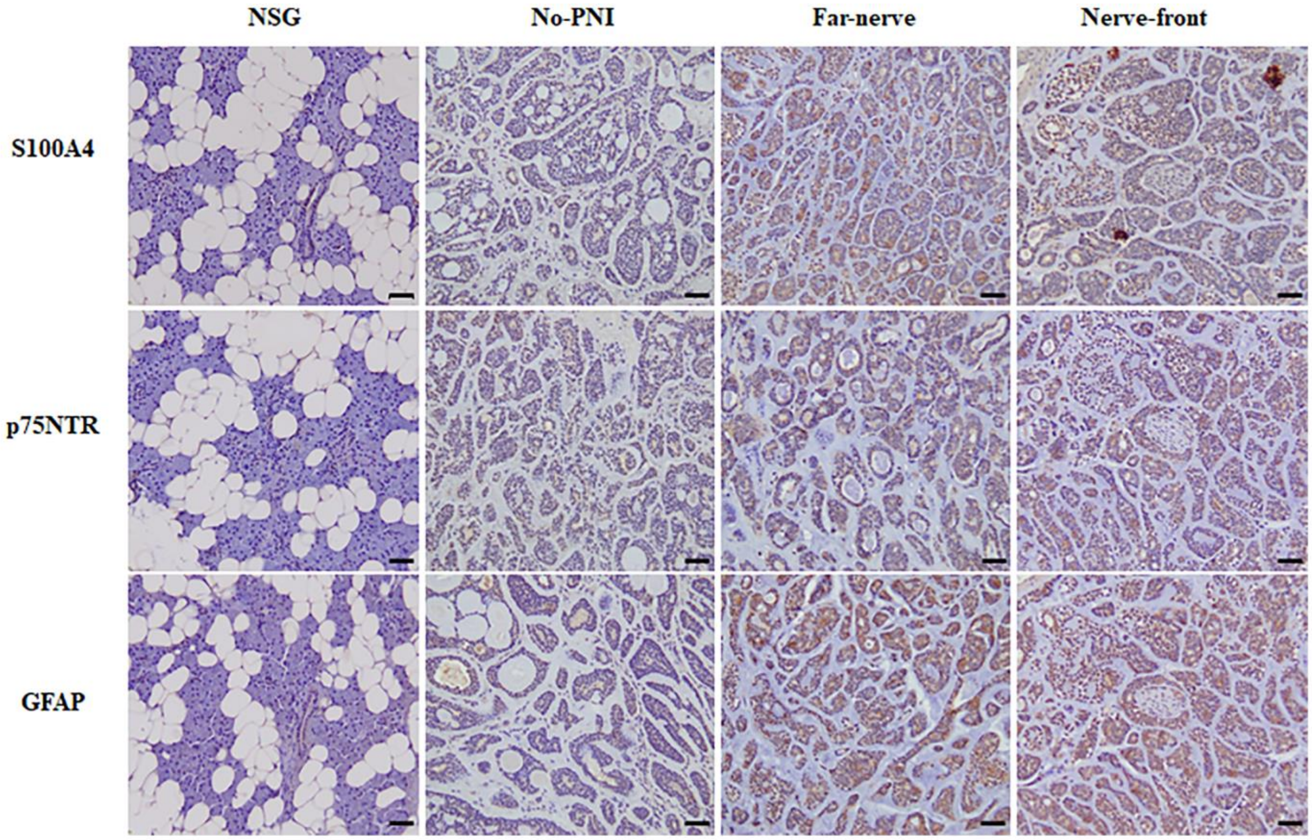

Supplementary Figure 1. Immunohistochemical staining of S100A4, p75NTR and GFAP in normal salivary gland (NSG), SACC without PNI (No-PNI), far away from nerve of SACC with PNI (Far-nerve), nerve invasion front of SACC with PNI (Nerve-front), (Bar: 50 μm).
