# Supplementary material for: CXCR5 induces perineural invasion of salivary adenoid cystic carcinoma by inhibiting microRNA-187
Source: Aging (Albany NY). 2021 Jun 10;13(11):15384–99. doi: 10.18632/aging.203097 (PMC8221347; doi:10.18632/aging.203097)
Supplement: Supplementary Table 1 [file aging-13-203097-s002.pdf]

## SUPPLEMENTARY TABLE

**Supplementary Table 1. The primer sequences of genes.**

| Gene | Primers (5'-3') |
|---|---|
| CXCR5-F | CGGCAGACACGCAGTTCCAC |
| CXCR5-R | ACGGCAAAGGGCAAGATGAAGAC |
| S100A4-F | GTACTCGGGCAAAGAGGGTG |
| S100A4-R | TTGTCCCTGTTGCTGTCCAA |
| P75NTR-F | CCTACGGCTACTACCAGGAT |
| P75NTR-R | TGGCCTCGTCGGAATACG |
| GFAP-F | CCGACAGCAGGTCCATGTG |
| GFAP-R | GTTGCTGGACGCCATTGC |
| GAPDH-F | CTTTGGTATCGTGGAAGGACTC |
| GAPDH-R | GTAGAGGCAGGGATGATGTTCT |
| Hsa-miR-370-F | CCAGGTCACGTCTCTGCAGTTAC |
| Hsa-miR-155-F | CGCGCGCTCCTACATATTAGCATTAAC |
| Hsa-miR-31-F | CCGTGCTATGCCAACATATTGCCAT |
| Hsa-miR-410-F | CGCGAATATAACACAGATGGCCTGT |
| Hsa-miR-187-F | CTCGTGTCTTGTGTTGCAGCC |
| Hsa-miR-126-F | CGCGCATTATTACTTTTGGTACGCG |
| Hsa-miR-224-F | GCGAAAATGGTGCCCTAGTGACTAC |
| Hsa-miR-205-F | CCGCGATTTCAGTGGAGTGAAGTTC |
